# Interplay of Postprandial Triglyceride-Rich Lipoprotein Composition and Adipokines in Obese Adolescents

**DOI:** 10.3390/ijms25021112

**Published:** 2024-01-16

**Authors:** Silvia García-Rodríguez, Juan M. Espinosa-Cabello, Aída García-González, Emilio González-Jiménez, María J. Aguilar-Cordero, José M. Castellano, Javier S. Perona

**Affiliations:** 1 Instituto de la Grasa-CSIC, 41013 Seville, Spain; silvia11_12@hotmail.com (S.G.-R.); juanmespinosa@ig.csic.es (J.M.E.-C.); jmcas@ig.csic.es (J.M.C.); 2 Department of Molecular Biology and Biochemical Engineering, Faculty of Experimental Sciences, University of Pablo de Olavide, 41013 Seville, Spain; agargon6@upo.es; 3 Instituto de Investigación Biosanitaria (ibs.GRANADA), 18016 Granada, Spain; emigoji@ugr.es; 4 Department of Nursing, Faculty of Health Sciences, University of Granada, 18071 Granada, Spain; mariajaguilar@telefonica.net

**Keywords:** obesity, adolescence, postprandial, triglyceride-rich lipoproteins, fatty acids, adipokines, ceruloplasmin

## Abstract

In the context of the alarming rise of infant obesity and its health implications, the present research aims to uncover disruptions in postprandial lipid metabolism and the composition of triglyceride-rich lipoproteins in obese adolescents. A double-blind, controlled clinical trial in the postprandial phase on 23 adolescents aged 12 to 16 years was carried out. Twelve participants were categorized as obese (BMI > 30 kg/m^2^ and percentile > 95) and 11 as normal-weight (BMI = 20–25 kg/m^2^, percentile 5–85). Blood samples were collected after a 12-h overnight fast and postprandially after consumption of a standardized breakfast containing olive oil, tomato, bread, orange juice, and skimmed milk. Obese adolescents exhibited elevated triglyceride concentrations in both fasting and postprandial states and higher TG/apo-B48 ratios, indicating larger postprandial triglyceride-rich lipoprotein (TRL) particle size, which suggests impaired clearance. Obese subjects also exhibited higher n-6 PUFA concentrations, potentially linked to increased TRL hydrolysis and the release of pro-inflammatory adipokines. In contrast, TRL from normal-weight individuals showed higher concentrations of oleic acid and DHA (n-3 PUFA), with possible anti-inflammatory effects. The results indicate an interplay involving postprandial TRL metabolism and adipokines within the context of adolescent obesity, pointing to potential cardiovascular implications in the future.

## 1. Introduction

Childhood and adolescent obesity, primarily stemming from inadequate diet and sedentary habits, is an alarming public health problem. Obesity has been related with the increased risk of insulin resistance, metabolic syndrome and its pathological complications, such as diabetes, hypertension, cardiovascular diseases, and certain types of cancer. In addition, a number of studies have shown that obese children and adolescents are more likely to remain obese into adulthood, further increasing the risk of developing these chronic diseases [1]. The problem has taken on epidemic proportions and the rates of overweight and obesity in children and adults continue to increase. More than four million people die each year from causes related to overweight or obesity, according to 2017 estimates of the global burden of disease [2]. Between 1975 and 2016, the prevalence of overweight or obesity in children and adolescents aged 5 to 19 more than quadrupled worldwide, from 4% to 18%. The vast majority of overweight or obese children live in developing countries, where the rate of increase exceeded that of developed countries by more than 30% [2]. Obesity is nowadays defined as a chronic, low-grade systemic inflammation state, in which the mechanisms responsible for resolving inflammation are impaired and there is a moderate but persistent increase in indicators of systemic inflammation [3]. It is now well-established that white adipose tissue is an important secretory organ metabolically active, with autocrine, paracrine and endocrine functions that can secrete a number of biologically active adipokines or adipocytokines, which are capable of modulating the inflammatory response [3].

Atherosclerosis is recognized as a chronic inflammatory pathology associated with the postprandial state, wherein alterations in lipids, the coagulation system, and endothelial function occur [4,5]. Several authors have underscored the impact of postprandial lipids and lipoproteins on acute inflammation and endothelial dysfunction, proposing that postprandial lipidemia may play a pivotal role in the development of atherosclerosis [6,7]. Because atherosclerotic alterations have their origin at relatively early ages, it would be of greatest interest to identify biochemical markers at an early age of life, to perform corrective treatment of harmful alterations in the lipid pattern.

Postprandial triglyceride-rich lipoproteins (TRL) have the ability to traverse the endothelial barrier, accessing the vascular subendothelial space where they interact with macrophages. These cells can take up TRL through various pathways, initiating foam cell formation, an early critical stage in atherogenesis. The extent and duration of the postprandial triglyceride (TG) response are influenced by diverse metabolic processes, including the rate of TG secretion from the intestine and liver, the activity of enzymes involved in TRL processing, and the speed at which TRL remnants are cleared through receptor-mediated pathways. These mechanisms are regulated by changes in particle size and composition, potentially impacting the TG incorporation into tissues [8].

Current evidence suggests that obese young people may exhibit disruptions in postprandial lipid metabolism, potentially contributing to cardiovascular complications. Moreno et al. [9] observed elevated circulating TG levels after an oral fat load in both obese and non-obese adolescents, with higher levels in those with bigger central fat distribution. Additionally, Umpaichitra et al. [10] identified postprandial hyperlipidemia in adolescents with type-2 diabetes and obesity. Furthermore, Su et al. [11] supported the association between elevated concentrations of circulating chylomicrons and cardiovascular disease risk in childhood obesity.

In a previous research, we demonstrated a strong correlation between insulin resistance and high circulating levels of some adipokines in adolescents, particularly in those who are obese [12]. In any case, information regarding the impact of adolescent obesity on postprandial TRL lipid composition and particle size, and its association with circulating concentrations of postprandial adipokines, remains scarce.

In light of the alarming increase in childhood and adolescent obesity and its associated health risks, the present study aims to investigate differences in postprandial TRL and circulating adipokines in obese adolescents, in comparison with non-obese individuals. We hypothesize that obesity in adolescence may disrupt postprandial lipid metabolism, affecting both the composition of postprandial TRL and the adipose tissue secretion of adipokines. By unraveling these connections, we aim to provide valuable insights into the mechanisms linking obesity, chronic low-grade systemic inflammation, and cardiovascular risk in adolescence, contributing to a broader understanding of preventive strategies and interventions for this growing public health concern.

## 2. Results

### 2.1. Baseline Characteristics of Participants

As displayed in Table 1, individuals of both study groups showed a similar average height, but they were clearly different in weight, and therefore also in body mass index (BMI), and in all dimensional anthropometric parameters. Furthermore, obese individuals exhibited higher systolic (SBP) and diastolic (DBP) blood pressure values. In all cases, the differences found were statistically significant. In addition, obesity caused higher levels of fasting insulin, which was also reflected in a higher homeostatic model assessment of insulin resistance (HOMA-IR) score and lower McAuley index. On the other hand, as expected, circulating TG are significantly more elevated in obese adolescents with respect to the normal-weight ones. By contrast, the concentration of HDL-cholesterol was lower, although within the normal range.

Regarding the values of inflammatory markers and adipokines measured at the beginning of the study, compared with normal-weight adolescents, the obese ones exhibited higher levels of the pro-inflammatory cytokines IL-1β, IL-6 and TNF-α. Likewise, obese individuals showed enhanced concentrations of C-reactive protein (CRP), leptin and ceruloplasmin. By contrast, the levels of MCP-1 and adiponectin were similar in both study groups.

### 2.2. Serum Triglycerides and ApoB48 during the Postprandial Phase

**Figure 1:**
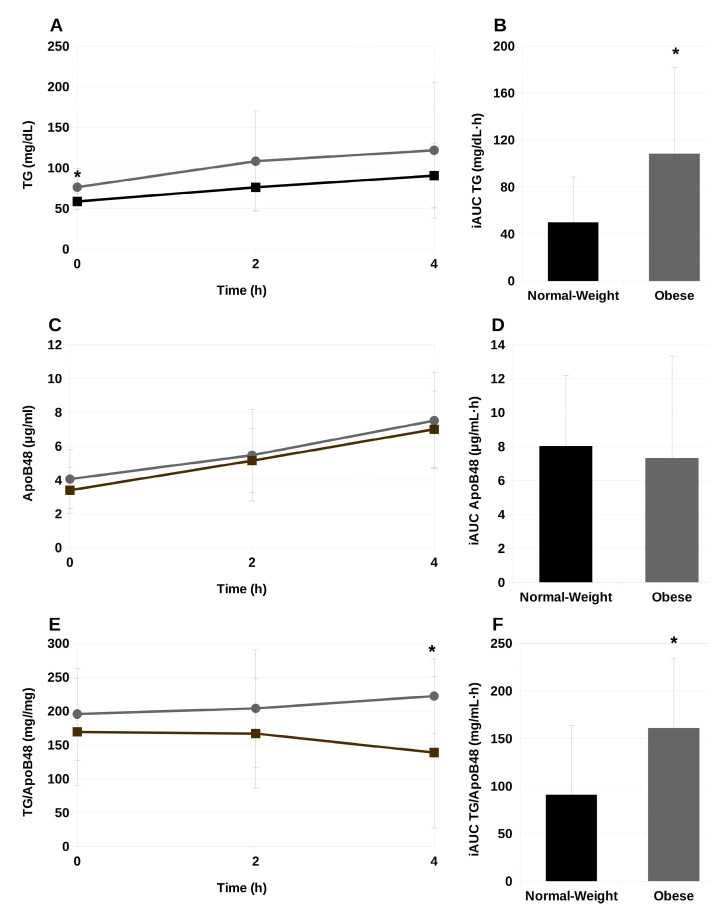
Postprandial serum triglycerides (**A**), apolipoprotein B48 (**C**) and triglyceride to apolipoprotein B48 ratio (**E**), and their corresponding incremental areas under the curve (iAUC; (**B**), (**D**) and (**F**), respectively) of normal-weight (square dots) and obese (circle dots) adolescents before (0 h) and after the intake of the experimental meal. TG, triglycerides; ApoB48, apolipoprotein B48; TG/ApoB48, triglyceride to apolipoprotein B48 ratio. Values expressed as mean ± SD. *, *p* < 0.05. Normal-weight, *n* = 11; Obese, *n* = 12.

Along the postprandial period the expected rise in the circulating TG levels followed an almost linear progression, at least until the fourth hour of this phase (Figure 1A). In the subjects of both study groups this evolution was similar, but in obese adolescents, the higher TG levels remained constant, in comparison with the normal-weight individuals. This difference was more remarkably evidenced by the incremental area under the curve (iAUC) for TG, being the difference significant (*p* < 0.05) between normal-weight and obese subjects (Figure 1B). Figure 1C displays the evolution of serum ApoB48, the specific apolipoprotein of chylomicrons. As well as in serum TG, a linear increment of serum ApoB48 in the subjects of both study groups was observed, with no significant differences among them, in the absolute values nor iAUC values (Figure 1C,D). The course of the TG/ApoB48 ratio is presented in Figure 1E, which reflects the higher TG content in sera of obese participants, a difference which became significant (*p* < 0.05) at 4 h postprandially (Figure 1E,F).

### 2.3. Association between Serum TG Concentrations and Biochemical and Anthropometric Parameters

TG were the only lipid species than changed throughout the postprandial state and baseline concentrations correlated significantly with some of the biochemical and anthropometric parameters analyzed, as shown in Table 2. In the normal-weight group fasting TG correlated only with Apo A-I and ceruloplasmin. In contrast, in obese individuals, TG correlated negatively with HDL-cholesterol and positively with lipoprotein (a), Apo B, BMI, waist circumference, hip circumference, leptin and ceruloplasmin. However, significance was lost when data were corrected for multiple comparisons.

### 2.4. Circulating Adipokines during the Postprandial Period

**Figure 2:**
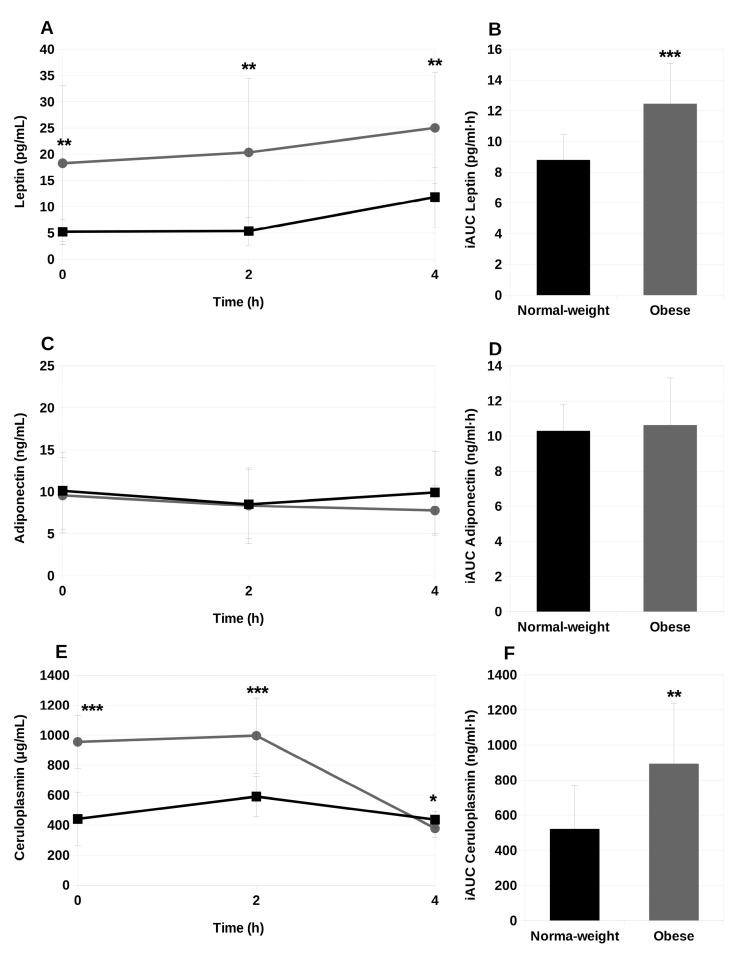
Postprandial serum leptin (**A**), adiponectin B48 (**C**) and ceruloplasmin (**E**) concentrations, and their corresponding incremental areas under the curve (iAUC; (**B**), (**D**) and (**F**), respectively) of normal-weight (square dots) and obese (circle dots) adolescents before (0 h) and after the intake of the experimental meal. Values expressed as mean ± SD. *, *p* < 0.05; **, *p* < 0.01; ***, *p* < 0.001 vs. normal-weight individuals. Normal-weight, *n* = 11; Obese, *n* = 12.

During the postprandial period, obese adolescents maintained significantly higher levels of serum leptin (*p* < 0.01) compared with normal-weight participants (Figure 2A), in a clear trend of sustained rise. Considering the iAUC for serum leptin in the 0–4 h postprandial lap, this difference among the study groups became highly significant (*p* < 0.001) (Figure 2B). By contrast, serum adiponectin contents remained practically unchanged throughout the postprandial phase, with no differences between the study groups (Figure 2C,D). In the case of ceruloplasmin, obese adolescents presented more than twice as higher levels compared with the normal-weight individuals at the baseline state (Figure 2E,F). At 2 h postprandially, this difference was maintained, and at 4 h after the experimental breakfast intake, the normal-weight adolescents showed slightly higher, though significant, contents of serum ceruloplasmin than the obese ones. In consequence, the iAUC for ceruloplasmin in obese adolescents was significantly higher (*p* < 0.01) compared to normal-weight adolescents.

### 2.5. Analysis of Fatty Acids in Triglycerides and Phospholipids Isolated from TRL

The fatty acid (FA) composition of TG isolated from TRL of the participants in the trial is displayed in Table 3. At baseline, TG in TRL particles from the obese individuals showed lower levels of 18:1 n-9 (oleic acid), 20:0 (eicosanoic acid) and 22:6 n-3 (docosahexaenoic acid, DHA). In contrast, the concentration of 18:2 n-6 (linoleic acid) and 20:2 n-6 (eicosadienoic acid) were higher than in normal-weight adolescents. Considering the different FA groups, both normal-weight and obese adolescents showed similar contents of saturated fatty acids (SFA), whereas in the obese participants the levels of n-6 polyunsaturated (n-6 PUFA) were significantly higher and those of monounsaturated (MUFA) and n-3 PUFA were lower than those in normal-weight adolescents.

Throughout the postprandial period, the differences in the baseline contents of 18:0 (stearic acid) and linoleic acid between obese and normal-weight subjects remained consistent. Additionally, significant differences were observed in the content of 14:1 n-5 (myristoleic acid), oleic acid and DHA contents at 4 h postprandially, being their levels in obese adolescents lower for the MUFA and higher for DHA, in comparison with the normal-weight subjects. In general, postprandial TRL obtained from obese adolescents presented lower contents of MUFA and higher of PUFA (n-6 and n-3).

Regarding the FA composition of phospholipids (PL) isolated from TRL, Table 4 shows that in the baseline state, obese adolescents presented lower contents of 14:0 (myristic acid), myristoleic acid, eicosanoic acid and 20:1 n-9 (eicosenoic acid), and higher levels of linoleic acid, eicosadienoic acid and 20:4 n-6 (arachidonic acid), in comparison with normal-weight individuals. Differences in TRL composition changed slightly in the postprandial phase, with lower contents of 16:1 n-7 (palmitoleic acid) and 18:3 n-3 (α-linolenic acid) in obese individuals at 2 h. At 4 h after the intake of the experimental meals, obese adolescents presented higher contents of archidic and arachidonic acids in their TRL-PL. In consequence, TRL derived from the normal-weight group were higher in MUFA and PUFA n-3, while those derived from the obese group were higher in PUFA n-6.

## 3. Discussion

Childhood and adolescent obesity, driven by poor diet and sedentary habits, is a significant public health threat, linked to increased risks of insulin resistance, metabolic syndrome, diabetes, hypertension, cardiovascular diseases, and certain cancers [1]. Obesity is now recognized as a chronic, low-grade inflammatory state, with white adipose tissue playing a crucial role by releasing adipokines that impact inflammation in various organs influencing the development of atherosclerosis [3]. Zilversmit proposed in 1979 that atherosclerosis is a postprandial phenomenon [13]. The formation of the atherogenic plaque is associated with postprandial states and involves disturbances in lipid metabolism [4,5]. Obese children exhibit postprandial lipid metabolism issues, potentially contributing to cardiovascular complications. The present, study aimed to investigate the composition of postprandial TRL in obese adolescents in comparison with normal-weight subjects, exploring lipid composition, particle size, and their association with adipokine concentrations. Given that atherosclerotic alterations originate at relatively young ages, it would be highly valuable to identify biochemical markers in early life, to facilitate corrective interventions against detrimental alterations in the lipid profile.

In the present work, we found greater TG concentrations in the serum of obese adolescents, compared to the normal-weight counterparts, both in the fasting and the postprandial states (Figure 1A,B), which agrees with the results reported before [9,10,14,15]. Most circulating chylomicrons and VLDL undergo partial lipolysis, making the total serum TG concentration a general, non-specific indicator of the total concentrations of remaining lipoproteins [16]. Elevated plasma TG levels have been associated with cholesteryl ester exchange mediated by cholesteryl ester transfer protein (CETP) [17], leading to the conversion of cholesteryl ester-enriched HDL into TG-rich HDL particles, ultimately decreasing HDL-cholesterol concentrations. Despite suggestions that a high-fat meal could lower postprandial HDL-cholesterol levels [18], various studies, including one utilizing olive oil as a fat source, demonstrated only modest alterations or slight increases in HDL-cholesterol levels in individuals with Type-2 diabetes [19,20].

In contrast, we did not find increased presence of apo B-48, the marker of the number of circulating chylomicrons and their remnants (Figure 1C,D). Apo B48 is the characteristic apolipoprotein of chylomicrons, and therefore, is present in postprandial TRL, which consist mainly in chylomicrons although the presence of VLDL cannot be discarded. These TRLs compete with hepatic VLDL containing Apo B100 in the lipolytic and remnant elimination pathways, causing a dynamic pool of circulating TG [16]. Dietary fat in chylomicrons accounts for about 80% of the increase in serum TG levels after a meal. An increase in Apo B48 during the postprandial phase would indicate an increase in the number of these postprandial TRL particles [21]. Current evidence suggests that obese individuals have higher serum apoB-48 concentrations [22,23]. In adolescents, the evidence is not so ample. Still, there are data reporting that adolescents with obesity had significantly higher concentrations of apoB48 compared to lean controls [24]. However, these were cross-sectional studies in which apoB48 levels were measured in the fasting state. In fact, and in agreement with our results, Mager et al. [25] were unable to find differences in apo-B48 concentrations in the postprandial period in the sera of children with nonalcoholic fatty liver disease or obesity compared to lean ones. On the other hand, Wang et al. [26] found apo-B48 concentrations two-fold higher in obese pre-pubertal children, compared to normal-weight ones. These authors suggested that insulin resistance may promote the overproduction of TRL and interfere with their clearance, resulting in elevated circulating apoB48 concentrations.

Despite not finding differences in apoB-48 concentrations, higher TG concentrations, led to higher TG/apo-B48 ratios (Figure 1E,F). We have proposed there is a robust association between the TG/apolipoprotein B (Apo B) ratio and the size of TRL particles in adult males with a healthy normal weight [13]. While this correlation was not evident in obese individuals, we believe that the TG/Apo B ratio could serve as a valuable means to gauge TRL size as it has been previously employed for this purpose and proved its reliability and convenience [27,28]. Therefore, we can assume that the higher TG/apo-B48 found in the present study, in terms of iAUC, may be related to higher TRL particle size in the postprandial state. Although we and others have suggested that larger particles are more prone to hydrolysis and clearance from blood [29], we did not observe a reduction in TG and apoB-48 serum concentrations throughout the postprandial period up to 4 h. This indicates that TRL remnants remain in the bloodstream for longer and suggests an impairment in their clearance. Certainly, impairment in lipoprotein metabolism due to insulin resistance can affect not only the production of TRLs but also their hydrolysis. In this regard, Rodríguez-Mortera et al. [24] pointed out that obese adolescents exhibit elevated levels of angiopoietin-related protein 3 (ANGPTL3) along with increased apo CIII, contributing to reduced lipoprotein lipase (LPL) mass and activity, the enzyme that hydrolyzes TG from TRL, releasing FA. Reduced LPL activity may lead to increased presence of TRL remnants in the bloodstream, which might have deleterious consequences in the formation of the atherosclerotic plaque.

The activity of LPL is influenced not only by ANGPTL3 but also by the FA composition of TRL. The enzyme can distinguish between substrates, demonstrating specificity in terms of both FA chain length and unsaturation [30]. Consequently, the FA composition of TRL plays a pivotal role in determining LPL activity and the formation of atherogenic TRL remnants. According to Sato et al. [30], LPL exhibits preference for SFA, followed by MUFA and PUFA. Therefore, TRL remnants tend to be enriched in PUFA that remain in the particle core. In the present study we found higher concentrations of linoleic, eicosadienoic and arachidonic acids, which are n-6 PUFA, in TRL obtained from obese individuals. This would be in agreement with higher TRL hydrolysis, release of SFA and MUFA and production of TRL remnants, supporting the idea that higher size particles are more efficiently hydrolyzed.

Considering linoleic acid as an essential FA that cannot be synthesized by humans, its presence in TRL must originate from the meal or from endogenous sources, both in PL and TG [31]. Since the content of linoleic acid in the experimental oil (olive oil) was relatively low, its presence in TRL suggests an endogenous origin. In addition, experimental meals were identical in both groups, so discrepancies in linoleic acid incorporation into nascent chylomicrons from enterocytes should be minimal. Therefore, differences in PL and TG may be attributed to the altered TRL metabolism in obese individuals via LPL. Consequently, we believe that, as discussed earlier, the more efficient hydrolysis of larger particles by LPL, releasing more SFA and MUFA while retaining more PUFA, contributes to the observed differences in TRL composition.

N-6 PUFA, specifically arachidonic acid and its substrate linoleic acid, are considered pro-inflammatory as they lead to the production of eicosanoids. These compounds include prostaglandins, prostacyclins, thromboxanes, and leukotrienes, and play a crucial role in regulating inflammatory and immune responses. The synthesis of eicosanoid products is controlled by enzymes such as cyclooxygenases (COX), lipoxygenases, and cytochrome P450s, with most arachidonic acid-derived eicosanoids being pro-inflammatory, except for prostaglandin E2 (PGE2) and lipoxins, which have anti-inflammatory effects [32,33]. Lab-made TRL particles incorporating n-6 PUFA induce a robust expression of COX-2 and subsequent prostanoid production in endothelial cells [34]. This activation is accompanied by the stimulation of ERK1/2 and p38MAPK, along with the suppression of basal and agonist-stimulated cGMP formation, indicating reduced nitric oxide (NO) production. Additional research has indicated that linoleic acid emerges as a significant byproduct during the co-incubation of TRL and LPL. This process not only produces a variety of oxidized free FA (FFA) but also triggers predominantly pro-inflammatory responses in arterial endothelial cells. These responses include the synthesis of cytokines and heightened expression of adhesion molecules [35].

In contrast, TRL isolated from normal-weight participants presented higher concentrations of oleic acid (MUFA) and DHA (n-3 PUFA), both in TG and PL. According to Margioris [36], the most important modulators of postprandial immune response appear to be PUFA, with n-3 PUFA suppressing postprandial inflammation, and n-6 PUFA promoting it. The molecular mechanisms suggested for the beneficial effect of n-3 PUFA on inflammation include the suppression of gene expression of proinflammatory cytokines, inhibition of adhesion molecule production in vascular endothelial cells, and induction of the release of the vasodilator NO, since n-3 PUFA can competitively substitute arachidonic acid in the PL of cell membranes.

Studies in the fat-1 transgenic mouse model, demonstrated that a decreased n-6/n-3 FA ratio reduces atherosclerotic lesions in apoE(−/−) mice [37]. Fat-1 mice express an n-3 FA desaturase that is capable of producing n-3 PUFAs from n-6 PUFAs and thereby has a ratio of n-6/n-3 FA close to 1:1 in tissues and organs. These animals were crossed with apoE (−/−) mice to develop an apoE(−/−)/fat-1 strain. When these mice were fed a Western-type diet, rich in n-6 PUFA, they developed less aortic lesions than the apoE (−/−) controls and exhibited higher production of inflammatory markers.

Unlike large chylomicrons and VLDLs that are hindered by their particle size, smaller TRL remnants can penetrate the arterial wall. The capacity of TRL remnants to transfer their lipid cargo to macrophages is a pivotal factor contributing to an elevated risk of atherosclerosis. Although remnant particles carry more TG than cholesterol, they can contain up to twice as much cholesterol per particle as LDL. Notably, TRL remnants are considered to be at least as atherogenic as LDL [38,39]. TRL remnants have the ability of transforming macrophages into foam cells by means of intracellular lipid accumulation, triggering the atherogenic process [40]. As a consequence, the cell acquires a pro-inflammatory state, with the release of inflammatory markers, such as cytokines. In this regard, chylomicron remnants have been shown to induce TG storage in J774 macrophages, with the enrichment of n-6 PUFA intensifying this effect compared to n-3 PUFA [41]. This was also true when THP-1 monocytes/macrophages treated with linoleic acid-rich TRL are compared with oleic acid-rich particles [42]. In the same cell line, stimulated with a phorbol ester (PMA) and oxidized LDL, Song et al. [43] found that incubation with n-6 and n-3 PUFA led to foam cell formation differently, depending on the ratio of these FAs. Low and middle n-6/n-3 PUFA ratios but not high ratios decreased cholesterol accumulation in the cells and the release of pro-inflammatory cytokines (IL-6 and TNFα). Therefore, TRL and their remnants, particularly when enriched with n-6 PUFA, may contribute to atherogenesis by inducing foam cell formation and a pro-inflammatory state, while MUFA and n-3 PUFA may have a potential protective effect. Indeed, the higher presence of n-6 PUFA in TRL from obese subjects might be associated with the elevated serum concentrations of IL-6, IL-1β and TNFα found in this group. Higher levels of these cytokines have been found in the blood of obese adolescents [44,45] and those with metabolic syndrome [46].

Remnant lipoproteins that undergo oxidative modifications play a significant role in atherogenesis due to their proinflammatory effects and uncontrolled uptake by scavenger receptors on macrophages. These lipoproteins also transport thrombogenic factors, activate monocytes, and promote endothelial cell death and smooth muscle cell proliferation, among other atherogenic characteristics. Elevated levels of Apo CIII in remnants may also contribute to atherogenic effects, as they have been shown to facilitate monocyte adhesion to endothelial cells and trigger inflammation through alternative inflammasome activation [39]. Remnants are susceptible to further modification due to their prolonged plasma residence time, potentially increasing their atherogenicity.

Adipokines, such as adiponectin and leptin, but also ceruloplasmin, have been linked to systemic inflammation, with adipogenesis contributing to the production of pro-inflammatory cytokines in obesity [47]. In fact, the leptin-adiponectin axis may contribute to increased inflammation and oxidative stress in individuals with metabolic syndrome [46]. There is controversy on the changes of plasma adiponectin concentrations in obese or diabetic subjects in the postprandial period, with authors showing a decrease [48], an increase [49] or no changes [50,51,52,53]. In obese pre-pubertal children, Gil-Campos et al. [51] found lower adiponectin levels, both in the fasting and the postprandial state. In this context, our results agree with those reported by Larsen et al. [54], who described an apparent postprandial suppression of adiponectin levels in normal-weight individuals in response to a fat meal, but noted that these effect was abolished in obese subjects. These authors suggested that adiponectin plays a regulatory action associated to postprandial TG clearance in the white adipose tissue, but that this complex and poorly understood regulatory mechanism may be overruled in obese individuals. Adiponectin appears to play a tuning role in free FA oxidation—especially in adipose tissue to accommodate storage of postprandial excess of TG—in enhancing FA oxidation in skeletal muscle and improving insulin sensitivity, which suppresses gluconeogenesis in the liver. In addition, attention has been focused on the fibroblast growth factor-21 (FGF-21)-adiponectin axis [55], which may be involved in the postprandial clearance of TRL fractions in healthy individuals [56], with adiponectin likely mediating this response. Therefore, it has been speculated that the unchanged postprandial levels of adiponectin in obese individuals after the experimental fat could be explained, considering that the FGF-21-adiponectin axis is overruled in obesity, is perhaps due to FGF-21 resistance [54].

On the other hand, postprandial leptin levels are consistently decreased in obese adults [57,58], but are in striking contrast with the increments found in adolescents [51], although there is little information available on the topic. Still, these results are in agreement with the ones we are presenting here. Interestingly, Larsen et al. [54] suggested that the impaired postprandial concentrations of adiponectin and leptin observed in obese individuals might contribute to delayed TG clearance. Regarding ceruloplasmin, to the best of our knowledge the postprandial concentrations of this copper-carrying protein has not been addressed so far, even in adults. In obese adolescents, we now show higher ceruloplasmin levels throughout the postprandial period, compared to normal-weight individuals. After a cross-sectional study on 976 adolescents, we suggested that ceruloplasmin might be a useful tool to identify individuals with metabolic syndrome and, thus, with a high risk of future cardiovascular disease [59]. In fact, in the preset study ceruloplasmin was the only parameter that correlated with serum TG levels both in normal-weight and obese adolescents.

This study reported some innovative findings on the complex interplay between postprandial lipid metabolism and inflammation, in the context of adolescent obesity and its potential future atherosclerotic complications. A strength of this study was that the results were obtained through the development of a double-blind, controlled clinical trial in the postprandial phase with 23 participants, where the energy intake provided by the experimental meal was standardized. However, there were also some limitations. First, a more frequent and longer blood-sampling time would provide more information on TG clearance and adipokine changes in response to the experimental meal. Second, we were unable to measure proinflammatory cytokines and NO in the postprandial phase due to insufficient serum aliquots, as most of the volume of these biological samples was used for TRL isolation and characterization. Repeatedly extracting substantial volumes of blood from a group of adolescents is understandably challenging. Thirdly, this analysis encompassed the entire populations of each study group, lacking subgroup analyses to assess the impact of participant characteristics, such as sex. However, we did not find significant differences between boys and girls for any of the baseline variables in the two groups of study. We agree that there is evidence indicating differences in glucose metabolism between sexes in people over 20 years of age. However, we have previously conducted extensive work on large groups of adolescents of the same age as the participants in the present study (12–16 years) and found no such differences in glucose, insulin, and HOMA-IR values between boys and girls [13,60]. It is also possible that glucose levels and HOMA-IR values were higher if they had been measured in plasma, but that would have affected both obese and normal-weight groups. Finally, a larger number of participants in future studies would increase the power to detect statistical differences in the serum levels of lipid metabolites, proinflammatory agents, and adipokines measured in this trial.

In summary, in this work we describe that obese adolescents exhibit higher TG concentrations both in the fasting and postprandial states, along with increased ceruloplasmin levels throughout the postprandial period. Despite no significant difference in apoB-48 concentrations were found, the higher TG/apo-B48 ratio suggested larger TRL particle size in the postprandial state, potentially indicating impaired clearance. These results may be related to differences observed in the FA composition of postprandial TRL between obese and normal-weight individuals. The higher presence of n-6 PUFA, particularly arachidonic acid, in particles obtained from obese subjects, suggested increased TRL hydrolysis, together with higher release of pro-inflammatory adipokines. In contrast, TRL from normal-weight individuals had higher concentrations of oleic acid (MUFA) and DHA (n-3 PUFA), potentially contributing to postprandial anti-inflammatory effects. Overall, the findings suggest a complex interplay between postprandial lipid metabolism, inflammatory markers, and adipokines in the context of adolescent obesity and its future cardiovascular implications.

## 4. Materials and Methods

### 4.1. Design Overview, Settings and Participants

The study was designed as a double-blind, controlled clinical trial in the postprandial phase, conducted at San Cecilio Hospital (Granada, Spain). Eligible participants were healthy adolescents attending schools in Guadix (Granada, Spain), aged 12 to 16 years, who did not suffer from any digestive or metabolic disorders, as confirmed from their electronic medical records. Participants were categorized as either obese or normal-weight based on the BMI score, following the criteria outlined by the Obesity Task Force as per Cole et al.’s guidelines [14]. Among the subjects, 12 met the criteria for obesity (BMI > 30 kg/m^2^ and percentile > 95), while 11 were classified as normal-weight (BMI = 20–25 kg/m^2^ and percentile range 5–85). The study received approval (25 January 2011) from the Institutional Committee on Human Research at Hospital San Cecilio in Granada, Spain, and written informed consent was obtained from the volunteers’ parents. All procedures adhered to the ethical standards for human experimentation set by the institution and the national guidelines, in compliance with the Helsinki Declaration of 1975 (revised in 2000). The trial was registered at clinicaltrials.gov (NCT01518803).

### 4.2. Intervention

A total of 23 volunteers that met the criteria for inclusion in the trial were examined. On test days, following a 12 h overnight fast, blood samples were collected from the cubital vein to determine general, liver, and kidney biochemical parameters. Subsequently, the participants ingested an experimental breakfast. The composition of the breakfast is common in Southern Spain and consisted of commercial olive oil (30 g), crushed natural tomato (20 g), three slices of bread (23.7 g/slice; Panrico línea, Adam Foods, Reinosa, Spain), orange juice (250 mL), and a glass of skimmed milk (200 mL), providing 418 kJ of energy. The olive oil consumed referred to as “Cardioliva”, was generously donated by Oleícola El Tejar S.C.A (El Tejar, Córdoba, Spain), while the other foods were purchased from the local market. After consuming the breakfast, blood samples were collected from the participants at 2 and 4 h postprandially. These time points were chosen based on previous studies showing that this period includes the relative maximum concentrations of serum TG [15].

### 4.3. Anthropometric Measures of Participants

Anthropometric assessments were conducted individually following the guidelines of the International Society for the Advancement of Kinanthropometry (ISAK) [16]. For body measurements, a TANITA^®^ BC-418MA body composition analyzer (West Drayton, UK) was used. Height was measured twice using a portable Seca 214* stadiometer, with an accuracy of 5 mm. Body mass index (BMI) was calculated by dividing weight in kilograms by the square of height in meters. Waist circumference (WC) was measured by placing an automatic roll-up measuring tape from Seca (with a precision of 1 mm) at the midpoint between the lowest rib and the upper edge of the iliac crest, at the end of a normal exhalation. Blood pressure (BP) readings were obtained using a calibrated aneroid sphygmomanometer along with a Littmann^®^ stethoscope (from Saint Paul, MN, USA), following internationally recognized recommendations [17].

### 4.4. Clinical Biochemistry of Participants

Blood samples were submitted to the Clinical Biochemistry and Analysis Laboratory of the Virgen del Rocío University Hospital for biochemical determinations. Serum glucose, total cholesterol, and triglycerides were determined using enzymatic colorimetric methods on a Roche/Hitachi System analyzer (Roche Diagnostic, Mannheim, Germany). Serum HDL cholesterol was measured through a direct enzymatic method (HDL-C-plus 2nd generation, Roche Diagnostics, Mannheim, Germany) on a Roche/Hitachi System analyzer (Roche Diagnostic), and LDL cholesterol was estimated using the Friedewald formula [18]. Total lipoprotein Apo B concentrations were determined through automated immunoturbidimetric assays (Tinaquant; Roche Diagnostics, Mannheim, Germany). Serum apoAI was assessed using rate immunonephelometry (BN II Nephelometer Dade Behring; Siemens Healthcare Diagnostics, Eschborn, Germany) automatically using the Roche/Hitachi Modular Analytics V1.1 system (Roche Diagnostics, Basel, Switzerland) located at Virgen del Rocío University Hospital (Seville, Spain). Insulin levels were measured using immunoassays (Abott Laboratories, Maidenhead, UK), and the HOMA-IR marker was calculated using the HOMA Calculator software v.2.2.3. (Diabetes Trial Unit, Churchill Hospital, Oxford, UK).

### 4.5. Inflammatory Markers Production

Interleukin (IL)-1β, IL-6, IL-10, tumor necrosis factor-alpha (TNF-α) and monocyte chemoattractant protein-1 (MCP-1) concentrations were measured in sera from participants at baseline using ELISA kits (Diaclone, Besancon, France), according to the manufacturer’s instructions. Absorbance at 450 and 630 nm were measured using a scanning multiwell spectrophotometer (Multiskan spectrum, Thermo-Fisher Scientific, Waltham, MA, USA). Absorbance at 630 nm was subtracted from that at 450 nm to correct optics imperfections. Finally, CRP was measured using ELISA kits (Proteintech, Manchester, UK) according to the manufacturer’s instructions. Samples with different concentrations of standards were analyzed to obtain calibration curves. Adiponectin, leptin and ceruloplasmin concentrations were measured in sera at 0, 2 and 4 h postprandially, using ELISA kits (AssayPro, St. Charles, MO, USA) according to the manufacturer’s instructions. Absorbance at 450 and 570 nm were measured using a scanning multiwell spectrophotometer (Multiskan spectrum, Thermo-Fisher Scientific, Waltham, MA, USA). Absorbance at 570 nm was subtracted from that at 450 nm to correct optics imperfections. Samples with different concentrations of standards were analyzed to obtain calibration curves.

### 4.6. Isolation and Characterization of Human Triglyceride-Rich Lipoproteins (TRL)

TRLs were isolated from blood aliquots drawn at 0, 2 and 4 h of the postprandial period by ultracentrifugation, as described by Cabello-Moruno et al. [19]. Briefly, TRLs were isolated from 4 mL of serum by salt-gradient ultracentrifugation with 6 mL of NaCl solution (density 1.006 g/mL) at 39,000 rpm, 16 h at 12 °C). Ultracentrifugation was performed using an SW 41 Ti rotor in a Beckman L8-70M preparative ultracentrifuge (Beckman Instruments, Palo Alto, CA, USA).

### 4.7. Lipid Extraction from TRL

The volume of TRL obtained from 4 mL of serum was adjusted to 1.5 mL final volume with KCl 0.1 M. Lipids were extracted as follows: Samples were placed into a conical tube and 5 mL of chloroform:methanol (2:1, *v*/*v*) was added. After shaking vigorously for 1 min, the samples were centrifuged at 3000 rpm for 10 min, obtaining three phases, of which the bottom one (chloroform phase) contained the lipids. This phase was collected and filtered on a Whatman #1 filter paper (Whatman Paper Ltd., Maidstone, UK) containing anhydrous sodium sulfate (Merck, Darmstadt, Germany), in order to retain any traces of water left, and placed in an evaporator tube. The extraction procedure was repeated in the aqueous phase, and the chloroform fractions combined and reduced to dryness in a rotary evaporator (Büchi Labortechnic AG, Flawil, Switzerland) at 40 °C. The residue was redissolved in 0.5 mL of chloroform:methanol (2:1, *v*/*v*) and kept at −20 °C in a nitrogen atmosphere until processing to prevent oxidation.

### 4.8. Triglyceride and Phospholipid Fatty Acid Composition

TG and PL fractions were isolated by solid phase extraction (SPE) using LC-Diol type cartridges (SupelcleanTM LC-Diol, Supelco, Bellefonte, PA, USA) of 3 mL capacity and 45 μm particle size. The cartridges were conditioned with hexane. Aliquots (200 μL) of sample was added and the TG fraction was eluted with two volumes of hexane:methylene chloride (9:1, *v/v*) (VWR Chemicals, Int., USA). Subsequently, the PL fraction was obtained by eluting with 3 mL of methanol and 3 mL of acetone. Both fractions were individually dried under a nitrogen stream and redissolved in 500 μL of chloroform:methanol (1:1, *v*/*v*). Fatty acids (FA) were derivatized to FAMEs following the hot methylation procedure on Annex B EU Regulation CE 2568/91 (4.2, IUPAC no 2.301). FAME obtained from TG and PL were analyzed by GC-FID, using a Hewlett-Packard 5890 Series II (Hewlett-Packard, Avondale, PA, USA) apparatus, equipped with a silica capillary BPX70 SGE, Analytical Science (Trajan, Victoria, Australia) column (10 m long; 0.1 mm inner diameter and 0.2 μm stationary phase thickness). Nitrogen was used as carrier gas, and the oven followed a temperature program (starting at 190 °C, and increasing at 5 °C/min until 250 °C, and remaining at this temperature until the end of the analysis). Injector and detector temperatures were kept constant at 270 °C. Chromatographic conditions and peak integration were performed by the ChemStation Rev. A-03-02 (Hewlett-Packard, Avondale, PA, USA) software. FAMEs were identified by comparison with the retention times of standards injected under the same conditions. Quantification was carried out by calculating the relative percentage of each FAME with respect to the total. Postprandial serum TG concentrations were determined using colorimetric enzymatic methods (Triglyceride GPO-PAP and Cholesterol CHOD-PAP, Roche Diagnostic, Mannheim, Germany) according to the manufacturer’s instructions.

### 4.9. Analysis of Total ApoB and Apo B48 Content in TRL

Total apo B was determined in TRL by immunoturbidimetry, using a BioSystems kit (BioSystems, Barcelona, Spain) according to the manufacturer’s instructions. Absorbance at 340 nm was measured with a Thermo Scientific Multiskan Spectrum spectrophotometer (Thermo Scientific, Waltham, MA, USA). A calibration curve was obtained with five different standard concentrations in the range 0–1.278 μg/μL. Apo B48 was determined using an ELISA kit (Abbexa Ltd., Cambridge, UK) according to the manufacturer’s instructions. Absorbance at 450 nm was measured with the Thermo Scientific Multiskan Spectrum spectrophotometer.

### 4.10. Statistical Analysis

The results are expressed as mean ± standard deviation (SD). Data analysis was performed using the GraphPad Prism^®^ 5 statistical package (GraphPad Software Inc., San Diego, CA, USA). The statistical significance was assessed using the unpaired or paired t-Student test, as appropriate. The statistical significance of baseline and postprandial levels of inflammation markers between the normal-weight and obese groups of adolescents was evaluated using one-way ANOVA, followed by the Tukey’s test. Correlations were carried out using Pearson’s test. Results were corrected for multiple comparisons using the Benjamini–Hochberg method when applicable. Since cytokine values were not normally distributed, they were log-transformed for statistical analysis. Differences with a *p*-value ≤ 0.05 were considered statistically significant.

## Figures and Tables

**Table 1:** Baseline serum biochemical and anthropometric measured in adolescent participants.

	Normal-Weight		Obese	
	Mean	SD	Mean	SD
Glucose (mg/dL)	59.8	6.8	60.0	12.2
Insulin (µIU/mL)	7.3	3.2	13.6	5.7 **
HOMA-IR	0.8	0.4	1.6	0.6 **
McAuley index	9.6	1.2	7.3	1.1 ***
HbA1c (%)	5.3	0.2	5.6	1.7
Cholesterol (mg/dL)	164.4	20.7	160.0	26.4
LDL-cholesterol (mg/dL)	83.0	18.6	87.8	20.3
HDL-cholesterol (mg/dL)	57.0	7.17	45.2	8.78 **
Lipoprotein (a)	57.3	37.3	76.9	53.2
Triglycerides (mg/dL)	58.7	10.2	76.3	22.8 *
Apolipoprotein A-I (mg/dL)	159.8	13.3	137.1	14.4 **
Apolipoprotein B (mg/dL)	53.8	8.3	60.5	10.8 ^1^
Weight (kg)	57.9	11.9	90.3	14.2 ***
Height (cm)	163.8	8.0	164.0	7.8
BMI (kg/m^2^)	21.4	3.0	33.4	3.0 ***
Systolic pressure (mmHg)	112.8	7.9	141.4	4.2 ***
Diastolic pressure (mmHg)	60.4	2.7	76.7	2.6 ***
Upper Arm Circumference (cm)	26.6	4.2	34.1	1.9 ***
Thigh Circumference (cm)	52.5	9.2	63.9	10.5 ***
Waist Circumference (cm)	79.7	14.2	100.7	8.6 **
Hip Circumference (cm)	91.7	10.5	107.8	7.8 ***
Tricipital fold (mm)	20.8	9.3	31.8	7.2 **
Bicipital fold (mm)	13.5	8.8	21.8	5.3 *
Subscapular fold (mm)	17.1	10.3	34.5	6.0 ***
Suprailiac fold (mm)	20.3	12.5	36.2	7.6 **
Calf Fold (mm)	18.9	10.2	31.4	8.6 **
Thigh Fold (mm)	31.1	13.34	43.4	6.2 *
C-reactive protein (mg/dL)	0.16	0.10	0.27	0.02 ***
MCP-1 (pg/mL)	140.4	40.5	146.7	65.3
IL-6 (pg/mL)	82.0	57.9	229.4	167.2 **
IL-1β (pg/mL)	261.2	182.8	426.6	167.1 *
TNF-α (pg/mL)	18.3	9.1	47.2	15.4 ***
Adiponectin (ng/mL)	10.3	1.5	10.6	2.7
Leptin (pg/mL)	8.8	1.7	12.5	2.6 **
Ceruloplasmin (μg/mL)	440.8	179.5	594.8	176.9 * ^1^

^1^ HOMA-IR, homeostasis model assessment of insulin resistance; HbA1c, glycated hemoglobin; MCP1, monocyte chemoattractant protein-1; IL-6, interleukin-6; IL-1β, interleukin-1β; TNF-α, tumor necrosis factor-α. Values expressed as mean ± SD. *, *p* < 0.05; **, *p* < 0.01; ***, *p* < 0.001 vs. normal-weight individuals. Normal-weight, *n* = 11; Obese, *n* = 12.

**Table 2:** Correlations between baseline TG concentrations and the serum biochemical and anthropometric measured in adolescent participants.

	Normal-Weight			Obese		
	r^2^	Crude *p*	Corrected *p*	r^2^	Crude *p*	Corrected *p*
Glucose (mg/dL)	−0.373	0.161	0.572	−0.116	0.360	0.411
Insulin (µIU/mL)	0.484	0.136	0.544	**0.609**	**0.040**	0.213
HOMA-IR	−0.266	0.245	0.490	0.341	0.139	0.262
McAuley index	−0.498	0.128	0.585	−0.452	0.130	0.260
HbA1c (%)	0.388	0.171	0.547	0.233	0.233	0.324
Cholesterol (mg/dL)	−0.515	0.066	0.528	0.337	0.142	0.252
LDL-cholesterol (mg/dL)	−0.541	0.078	0.499	0.481	0.057	0.182
HDL-cholesterol (mg/dL)	−0.525	0.060	0.640	**−0.405**	**0.026**	0.208
Lipoprotein (a) (mg/dL)	0.110	0.393	0.434	**0.869**	**0.006**	0.192
Apolipoprotein A-I (mg/dL)	**−0.563**	**0.045**	0.720	0.110	0.292	0.346
Apolipoprotein B (mg/dL)	−0.162	0.327	0.419	**0.503**	**0.048**	0.171
BMI (kg/m^2^)	0.001	0.928	0.928	**0.666**	**0.013**	0.208
Systolic pressure (mmHg)	0.198	0.292	0.467	−0.464	0.065	0.189
Diastolic pressure (mmHg)	0.116	0.375	0.429	−0.436	0.078	0.192
Upper Arm Circumference (cm)	−0.123	0.368	0.436	−0.444	0.074	0.197
Thigh Circumference (cm)	−0.217	0.274	0.516	−0.239	0.227	0.363
Waist Circumference (cm)	0.209	0.281	0.500	**0.506**	**0.047**	0.188
Hip Circumference (cm)	0.206	0.284	0.478	**0.617**	**0.016**	0.171
Tricipital fold (mm)	−0.279	0.217	0.534	0.220	0.246	0.328
Bicipital fold (mm)	−0.009	0.490	0.506	0.206	0.260	0.333
Subscapular fold (mm)	−0.106	0.324	0.432	−0.190	0.277	0.341
Suprailiac fold (mm)	−0.179	0.310	0.431	0.027	0.467	0.482
Calf Fold (mm)	−0.130	0.360	0.443	−0.113	0.363	0.401
Thigh Fold (mm)	−0.264	0.230	0.491	0.371	0.118	0.270
C-reactive protein (mg/dL)	0.444	0.189	0.550	−0.291	0.231	0.336
MCP-1 (pg/mL)	0.068	0.426	0.454	0.411	0.119	0.254
IL-6 (pg/mL)	0.740	0.103	0.549	0.757	0.227	0.346
IL-1β (pg/mL)	0.242	0.301	0.459	0.023	0.481	0.481
TNF-α (pg/mL)	0.570	0.215	0.573	0.233	0.425	0.453
Adiponectin (ng/mL)	0.181	0.308	0.448	0.377	0.142	0.239
Leptin (pg/mL)	0.349	0.222	0.507	**0.584**	**0.030**	0.192
Ceruloplasmin (mg/mL)	**0.747**	**0.017**	0.544	**0.572**	**0.042**	0.192 ^1^

^1^ HOMA-IR, homeostasis model assessment of insulin resistance; HbA1c, glycated hemoglobin; MCP1, monocyte chemoattractant protein-1; IL-6, interleukin-6; IL-1β, interleukin-1β; TNF-α, tumor necrosis factor-a. Normal-weight, *n* = 11; Obese, *n* = 12. Significant results are highlighted in bold (Crude *p*). *p*-values were corrected using the Benjamini–Hochberg method (Corrected *p*).

**Table 3:** Fatty acid composition of triglycerides in fasting and postprandial triglyceride-rich lipoproteins isolated from normal-weight and obese adolescents before (0 h) and after the intake of the experimental meal (mg/100 mg).

	0 h				2 h				4 h			
	Normal-Weight		Obese		Normal-Weight		Obese		Normal-Weight		Obese	
	Mean	SD	Mean	SD	Mean	SD	Mean	SD	Mean	SD	Mean	SD
14:0	1.41	0.91	1.25	0.60	1.13	0.57	1.15	0.36	1.59	0.17	1.47	0.26
14:1 n-5	0.46	0.32	0.22	0.25	0.59	0.73	0.26	0.23	0.48	0.13	0.24	0.08 ***
16:0	20.33	2.38	21.57	2.87	20.60	2.13	21.04	3.10	19.55	1.47	21.20	2.83
16:1 n-7	4.66	1.60	4.14	0.88	3.76	1.14	3.92	0.81	3.51	0.71	3.87	0.62
18:0	6.86	3.64	4.25	1.53 *	7.87	5.16	4.39	1.45 *	5.79	1.30	4.16	0.86 **
18:1 n-9	38.57	6.61	38.09	3.64	40.52	7.34	40.70	4.48	44.28	3.53	40.49	3.98 *
18:2 n-6	19.28	4.62	23.73	4.94 *	18.26	3.52	22.01	3.25 *	18.17	1.77	20.91	2.23 **
18:3 n-6	0.66	0.71	0.79	0.28	0.49	0.30	0.65	0.26	0.54	0.15	0.77	0.22
18:3 n-3	0.74	0.33	0.66	0.27	0.83	0.57	0.56	0.18	0.58	0.13	0.73	0.22
20:0	0.60	0.52	0.39	0.19	0.68	0.63	0.42	0.20	0.35	0.08	0.38	0.13
20:1 n-9	1.19	0.78	0.54	0.62 *	1.38	1.73	0.54	0.66	0.27	0.08	0.34	0.10
20:2 n-6	0.32	0.14	0.49	0.16 *	0.26	0.11	0.44	0.11	0.17	0.05	0.30	0.09
20:4 n-6	2.07	0.50	2.59	0.50	1.92	0.55	2.20	0.33	1.57	0.23	1.49	0.18
20:5 n-3	0.55	0.22	0.47	0.20	0.72	0.61	0.36	0.22	2.24	0.23	2.41	0.28
22:6 n-3	2.30	0.86	1.36	0.17 **	1.32	0.23	1.53	0.21	0.90	0.18	1.23	0.26 **
SFA	29.20	5.48	27.46	4.24	30.28	7.25	27.00	4.23	27.29	2.16	27.21	3.22
MUFA	44.98	5.19	42.99	2.99	46.28	5.29	45.39	4.12	48.55	3.07	44.95	3.68 *
PUFA n-6	22.33	5.32	27.59	4.99 *	20.93	3.88	25.30	3.41 **	20.45	1.79	23.47	2.22 **
PUFA n-3	3.59	1.28	2.49	0.70 *	2.87	1.41	2.44	0.51	3.72	0.22	4.37	0.32 *** ^1^

^1^ Values expressed as mean ± SD. *, *p* < 0.05; **, *p* < 0.01; ***, *p* < 0.001 vs. normal-weight individuals. Normal-weight, *n* = 11; Obese, *n* = 12.

**Table 4:** Fatty acid composition of phospholipids in fasting and postprandial triglyceride-rich lipoproteins isolated from normal-weight and obese adolescents (mg/100 mg).

	0 h				2 h				4 h			
	Normal-Weight		Obese		Normal-Weight		Obese		Normal-Weight		Obese	
	Mean	SD	Mean	SD	Mean	SD	Mean	SD	Mean	SD	Mean	SD
14:0	1.96	1.21	1.09	0.62 *	1.36	1.15	0.94	0.54	2.14	0.30	1.99	0.52
14:1 n-5	1.54	1.11	0.74	0.72 *	0.32	0.21	0.50	0.36	0.37	0.16	0.35	0.08
16:0	25.02	6.10	25.33	6.07	23.82	3.36	24.10	2.39	21.92	1.01	20.54	2.03
16:1 n-7	3.03	2.31	2.62	1.88	4.21	2.63	2.48	1.43 **	1.88	0.18	1.97	0.18
18:0	18.59	5.41	19.16	2.56	18.46	1.72	17.73	3.37	15.59	1.50	15.05	1.65
18:1 n-9	17.31	6.03	15.17	2.92	17.11	6.05	16.84	6.41	25.60	1.46	26.11	1.84
18:2 n-6	11.95	3.06	15.68	3.60 *	13.99	5.44	16.36	3.62	19.25	1.36	18.19	1.32
18:3 n-6	0.39	0.23	0.50	0.25	0.47	0.27	0.35	0.16	1.17	0.13	1.08	0.10
18:3 n-3	2.61	1.99	1.86	1.32	3.48	2.31	1.83	1.33 *	0.28	0.09	0.35	0.10
20:0	1.58	1.32	0.69	0.66 *	0.43	0.21	0.79	0.76	0.18	0.06	0.26	0.08 *
20:1 n-9	2.48	1.69	1.16	0.73 *	1.00	0.74	1.41	1.04	1.28	0.29	1.21	0.24
20:2 n-6	1.79	0.79	2.90	1.23 *	3.53	2.03	2.70	1.11	2.03	1.15	2.13	0.63
20:4 n-6	6.28	2.50	8.37	2.79 *	7.24	3.53	7.73	2.11	5.48	1.04	7.52	0.68 ***
20:5 n-3	1.81	1.63	1.11	0.84	0.90	0.81	1.28	0.95	1.28	0.21	1.51	0.39
22:6 n-3	3.69	1.65	4.29	1.54	3.77	1.36	4.96	1.85	1.56	0.27	1.75	0.30
SFA	47.16	10.40	45.49	7.12	44.08	3.23	43.56	3.33	39.82	1.47	37.85	2.08
MUFA	24.32	5.80	19.31	3.85 *	22.55	6.59	21.23	5.06	29.13	1.48	29.64	1.82
PUFA n-6	20.41	5.24	27.91	7.31 **	25.23	8.13	27.14	5.31	27.93	1.21	28.91	1.10
PUFA n-3	8.22	4.53	5.11	2.52 *	8.14	2.68	8.07	2.60	3.12	0.25	3.61	0.41 ^1^

^1^ Values expressed as mean ± SD. *, *p* < 0.05; **, *p* < 0.01; ***, *p* < 0.001vs. normal-weight individuals. Normal-weight, *n* = 11; Obese, *n* = 12.

## Data Availability

The datasets generated during the trial are available from the corresponding author upon reasonable request.

## Abbreviations

ANGPTL3	angiopoietin-related protein 3
Apo B	Apolipoprotein B
Apo CIII	apolipoprotein CIII
Apo E	Apolipoprotein E
AUC	area under the curve
BMI	body mass index
CETP	cholesteryl ester transfer protein
COX	cyclooxygenase
CRP	c-reactive protein
DBP	diastolic blood pressure
DHA	docosahexaenoic acid
FA	fatty acids
HbA1c	glycated hemoglobin
HDL	high-density lipoprotein
HOMA-IR	homeostasic model assessment of insulin resistance
iAUC	incremental area under the curve
IL	interleukin
LDL	low-density lipoprotein
LPL	lipoprotein lipase
MCP-1	monocye chemoattractant protein-1
MUFA	monounsaturated fatty acids
NO	nitric oxide
PGE2	prostaglandin E2
PL	phospholipids
PMA	phorbol 12-myristate 13 acetate
PUFA	polyunsaturated fatty acids
SBP	systolic blood pressure
SFA	saturated fatty acids
TG	Triglycerides
TNF-α	tumor necrosis factor alpha
TRL	Triglyceride-rich lipoprotein
